# The influence of perceived behaviour control, attitude and empowerment on reported condom use and intention to use condoms among adolescents in rural Tanzania

**DOI:** 10.1186/s12978-015-0097-5

**Published:** 2015-11-13

**Authors:** Albino Kalolo, Stephen Matthew Kibusi

**Affiliations:** 1 Department of Community Health, St. Francis University college of Health and Allied Sciences, P.O.Box 175, Ifakara, Tanzania; 2 School of Nursing and Public Health, College of Health Sciences, The University of Dodoma, P.O. Box 395, Dodoma, Tanzania

**Keywords:** Safe sex behaviour, Rural school adolescents, Theory of planned behaviour, Empowerment model of health promotion, Tanzania

## Abstract

**Background:**

Despite the declining trends of Human immunodeficiency virus (HIV) infection in Sub-Saharan Africa (SSA), unsafe sexual behaviours among adolescents still represent a public health challenge. It is important to understand factors acting at different levels to influence sexual behaviour among adolescents. This study examined the influence of perceived behaviour control, subjective norms, attitudes and empowerment on intention to use condoms and reported use of condoms among adolescents in rural Tanzania.

**Methods:**

We used a questionnaire to collect data from 403 adolescents aged 14 through 19 years from nine randomly selected secondary schools in the Newala district located in the Southern part of Tanzania. The self-administered questionnaire collected information on sexual practices and factors such as attitudes, subjective norms, perceived behaviour control and empowerment. Binary logistic regression was performed to identify factors associated with intention to use and reported use of condoms.

**Results:**

Sexually active adolescents constituted 40.6 % of the sample, among them 49.7 % did not use a condom at last sexual intercourse and 49.8 % had multiple sex partners. Many (85 %) of sexually active respondents had their sexual debut between the ages of 14 to 17 years. Girls became sexually active earlier than boys. Perceived behaviour control predicted intentions to use condoms (AOR = 3.059, 95 % CI 1.324-7.065), thus demonstrating its importance in the decision to use a condom. Empowerment (odds ratio = 3.694, 95 % CI 1.295-10.535) and a positive attitude (AOR = 3.484, 95 % CI 1.132-10.72) predicted reported condom use, thus turning the decision to actions. Subjective norms had only indirect effects on intention and reported use of condoms.

**Conclusion:**

The findings suggest that unsafe sex practices are prevalent among school adolescents in rural areas of Tanzania. Perceived behaviour control and positive attitudes predict intensions to use condoms whereas empowerment predicts reported condom use. The findings may imply that safe sex promotion interventions that simultaneously address socio-cognitive and ecological determinants of sexual behaviours may improve adolescents’ safe sex behaviours.

## Background

The risk of acquiring HIV infection, STIs and early or unintended pregnancy is increased by unsafe sexual behaviours [1–5]. Unsafe sexual behaviuors such as little use of condoms and having multiple sexual partners present a persistent challenge in the fight against the spread of STIs and HIV infection [3, 4, 6, 7]. Adolescents have been at the center of unsafe sexual practices and thus continue to suffer from negative consequences of the behaviours [7–9]. Statistics on the burden of HIV infection show that among the 36.9 million living with HIV /AIDS as of 2014, young people aged between 15–24 years accounted for 42 % of new HIV infections [10]. Sub-Saharan Africa (SSA) carries 71 % of the burden [11]. The burden of early or unintended pregnancy continues to grow in SSA [12].

In Tanzania, by the end of 2013, the proportion of young people aged between 15–24 years living with HIV/AIDS stood at 11.2 % [13] .Yet only 43.4 % of adolescents aged 15–24 years had comprehensive knowledge of HIV/AIDS including knowing that consistent use of a condom during sexual intercourse and having just one uninfected faithful partner can reduce the chance of getting the AIDS virus, knowing that a healthy-looking person can have the AIDS virus, and being able to reject the most common local misconceptions about transmission or prevention of HIV/AIDS [13, 14]. Up to 32 % of the adolescent girls and 34.3 % of the boys aged 15–19 years are sexually active before they reach 15 years of age. Girls in rural areas are more likely to start sex before 15 years than their counterparts in urban areas. Condom use in this group is low with only 55.7 % of the adolescents reporting condom use at last higher-risk sex defined as sexual intercourse with a non-marital, non-cohabiting partner [14]. Up to 1.9 % of girls and 6.8 % of boys have multiple sexual partners and 44 % of them are either mothers or fathers by the age of 19 years [14].

To address the challenge of unsafe sexual behaviours among adolescents, countries have adopted various strategies that target adolescents in school and out of school [13–15]. School based-HIV interventions have been implemented [1, 16, 17] and recommended for scale up as they represent an important strategy to reach children and young people in large numbers [18]. In Tanzania, to address HIV transmission among school adolescents, sexuality education and HIV prevention interventions have been included as part of the school curriculum [14, 19].

Despite these efforts, unsafe sexual behaviours among school adolescents are still prevalent [1, 4, 6, 16–19]. Evaluations of the school-based interventions in poor-resource settings show impact of the interventions on increasing knowledge and changing attitudes towards safe sex behaviours but there is limited evidence about the impact of these interventions on actual practices of safe sexual behaviours [13, 23].

Sexual behaviour of adolescents is shaped by multiple factors [1, 4, 24] which either act at individual level such as knowledge and attitudes or those which result as a consequence of the surrounding environment (ecological factors) such as availability of condoms and policies that create favourable environment to use condoms [20, 24–26]. In an attempt to understand sexual behaviour several theories and models have been applied in past research. These theories and models have mainly been socio-cognitive [27–29] and ecological models [25] . Most of the existing evidence is devoted to isolated theories or combination of theories that target a group of factors which act at the same level and therefore they may not be sufficient to understand the complexities of the sexual behaviours among adolescents. Studies in Tanzania and elsewhere in SSA, have focused much on socio-cognitive theories alone, such as the Health Belief Model [21, 26] and the Theory of Planned Behaviour (TPB) [15, 30]. In order to understand the complexities of sexual behaviour and thereby device comprehensive programs to improve safe sexual behaviour among adolescents, there is a need for integrated approach.

The TPB is mainly a socio-cognitive theory that provides a detailed understanding of the influence of individual’s attitudes, subjective norms and perceived behaviour control on the intentions to act [31]. Behavioural intentions indicate how hard people are willing to try and how much effort people plan to exert toward performance of a given behaviour. In addition, Ajzen [28] proposed that perceived behavioural control could also predict behaviour directly when behaviour is not under complete volitional control and when perceptions of control are realistic. Literature has documented the link between attitudes and sexual behaviour outcomes [32]. The influence of subjective norms on sexual behaviours is well documented [33]. Ajzen’s construct of perceived behavioural control, which is similar to Bandura’s [27, 34] construct of self-efficacy has also been found to impact sexual behaviour outcomes [35, 36]. This theory has been widely applied to design interventions and guide studies in safe sexual behaviours among adolescents of sub-Saharan Africa [20, 22, 30, 37–40].

The empowerment model of health promotion (EHP)[41] proposes the use of bottom up, non-directive and client centred approaches to influence individual choices regarding healthy behaviours and creation of health public policy. It supports the idea of empowering adolescents through participatory, face to face encounters and not merely by providing information but rather seeking to empower choices by building individual capacity (self-empowerment).Youth empowerment is attained through such methods as peer resistance trainings, peer leadership programs, life skills trainings, participation in youth led organisations or groups and the active involvement of youth in decision making on health issues affecting their lives .The model puts emphasis on the power of healthy public policy and health education in influencing healthy behaviours. The approach is seen as central to shifting from information (sermons) to empowerment thus facilitating actual performance of behaviours [41, 42]

In the present study we combined the constructs from TPB [43] and EHP[41] to study sexual behaviours of school adolescents. The constructs are combined to provide a comprehensive understanding of the factors that affect sexual practices. We postulate that socio-cognitive (individual) factors are important in decision-making to adopt safe sexual behaviour, whereas ecological factors support the execution of the decision. The TPB sheds more light on individual factors whereas the EHP helps to understand the wider ecological factors that influence sexual practices. In this study we did not aim to asses any specific intervention or assessing the validity of the two theories but the study findings may offer a challenge to the existing safe sex interventions, specifically so for interventions targeting school adolescents.

This study aimed to describe the factors influencing sexual behaviours among adolescents residing in generally rural areas of Tanzania using a framework that combines socio-cognitive and ecological constructs. Specifically we aimed to (1) determine the extent of unsafe sexual practices of adolescents (2) determine the correlation between the predictor variables i.e., perceived behaviour control, subjective norms, attitudes and empowerment (3) determine the influence of perceived behavioural control, attitudes and empowerment on intention to use condoms and (4) determine the influence of perceived behavioural control, attitudes and empowerment on reported use of condoms.

## Methods

### Study settings

This study was conducted from May to August 2010 in Newala district located in the Mtwara region of Tanzania. At the time of data collection, the district counted a population of 183,930 with 84,914 men and 99,016 women and it was geographically divided into 3 divisions, 21 wards and 155 registered villages .The district had 26 secondary schools with an estimate of 8169 students. The current population stands at 205,492 (2012 census) with a growth rate of 1.2 % and the district has further been divided to include 5 divisions, 28 wards and 155 registered villages [44]. The HIV prevalence in Tanzania was 5.7 % at the time we conducted the study and it has been recorded to decline especially in young people aged 15–24 years [45]. The prevalence of HIV infection in the district stood at 3.1 % (antenatal care proxy) [46]. Secondary education in Tanzania consists of six years of schooling divided into two levels of four years (Ordinary level) and two years (Advanced level) as described in other studies [19, 26]. Only Ordinary level students were included in the present study.

### Study design

This study relied on a cross-sectional analysis of data collected from school adolescents of the Newala district. The essential features of the study include: (1) sampling of the secondary schools located in the district (2) Sampling the adolescents to participate in the study (3) Data collection using a self-administered questionnaire and (4) Conducting analysis of the collected data.

### Study population and sampling procedures

Sampling was done using multistage cluster sampling procedure. Twenty six O-level secondary schools were considered. Thereafter schools were stratified according to geographical divisions and nine schools were selected, three from each of the divisions. The schools consisted of a total population of 4392 students with 2126 males and 2266 females.

Within selected schools, we intended to invite for interview all students in all classes aged between 14 through 19 years. In this study, 403 study students filled a self-administered questionnaire. This sample size was adequate as it exceeded the calculated sample size of 389 students with an assumption of 80 % power, 42 % of youth who use condoms in previous studies [8] and an estimated error margin of 0.05.

### Data collection instrument and procedures

Primary data were collected using a pre-tested self-administered questionnaire. The questionnaire was designed after considering recommendations from previous researchers on TPB [47, 48] and the ecological perspectives [49, 50].The questionnaire collected information related to socio-demographic variables, sexual practices, knowledge on safe sex behaviours, attitudes, subjective norms and behaviour control. The questions were presented in one of the three formats: yes or no, Likert scale questions with responses ranging from three (agree, uncertain, disagree) or five categories (totally agree, agree, uncertain, disagree, and totally disagree) responses. The questionnaire was administered within the classroom by a trained research assistant. Research assistants were introduced to students by a teacher who later on was asked to leave the classroom to give students more freedom and privacy. Participation was voluntary and the questionnaire was anonymously filled and participants had an opportunity to withdraw at any point without being asked any questions The questionnaire was translated from English to Kiswahili (the lingua franca in Tanzania) to facilitate understanding of the items by the adolescents.

### Variables and measures

Sexual behaviours were measured by responses from questionnaire presented in binary (yes/no) or multiple responses depending on the variable measured. Behavioural variables consisted of age at sexual debut (14 years of age or younger, 15 years of age or older); number of lifetime sex partners (only one, two, three to five, six or more); use of condom in last risky sex(“yes,” used condom; “no,” did not use condom). Intention to use condoms was assessed by a statement “*I intend to use a condom next time I have sexual intercourse”* (“yes,” intends to use a condom; “no,” does not have intention to use a condom).Behavioural variable were considered as dependent variables for this study.

The independent variables were a combination of attitudes, subjective norms, perceived behaviour control and empowerment constructs that originate from TPB and EHP.

***Attitudes*:** defined as a person’s overall evaluation of behaviours related to and performing safe sex (consistent condom use during penetrative sexual intercourse, reduction of sex partners and delayed age at first sex (and secondary sexual abstinence). These were assessed by four items on one’s evaluation and feelings on the benefits of safe sex (two items) and related sub-behaviours of practising safe sex (two items) (e.g. *Condoms protect against HIV infection*). The expected response were Likert scale 1–5 (from totally agree to totally disagree).

***Subjective Norms***: refer to belief that specific referents think that the school adolescents should perform safe sex to protect themselves from sexually transmitted infections, HIV infection and unwanted pregnancies and the individual’s motivation to comply with referents. In this study, important referent was defined to mean parents, friends, teachers and religious leaders. Subjective norms were assessed by using ten items categorized as peer pressure from friends towards or against safe sex behaviours. We used statements such as *“I agree with my religious leaders’ opinion that I should not use condoms when having sexual intercourse* or *I agree with the opinion of my friends that I should use condoms when having sex”*. The expected responses were Likert scale 1–5 (from totally agree to totally disagree).

***Perceived behavior control****:* refer to adolescent’s perceptions of his/her ability to practice safe sex. In line with Ajzen’s work [47], perceived behavioral control was assessed with two components: perceived self-efficacy (close to Bandura’s [27] construct of self-efficacy, which has stronger path with intention than the behaviour) and perceived controllability (that has stronger path or link with behaviour than intention .We used statements such as *“It is mostly up to me whether I decide to use a condom or not”*. The expected responses were Likert scale 1–5 (from totally agree to totally disagree).

***Empowerment****:* refers to adolescent’s ability to taking a lead in safe sex promotion in school and/or out of school and control of the surrounding environment in the search for practicing health life styles, i.e., safe sex behaviors. We conceptualized empowerment as a two way process that involve conscious raising (self-empowerment) and at presence (or creation) of enabling environment to support performance of safe sexual behaviors. Empowerment was measured by asking adolescents’ involvement participatory health promotion programs in and out of school environment. The questions focused on their participation in life skills training sessions on sexual and reproductive health, peer education, music and sports festivals with safe sex promotion themes. In addition, adolescents were asked about their experiences with user friendliness of sexual and reproductive health services in their area and the policies that promote safe sexual behaviours. Their responses to these questions were binary i.e., “yes” or “no”.

### Data analysis

**Fig. 1:**
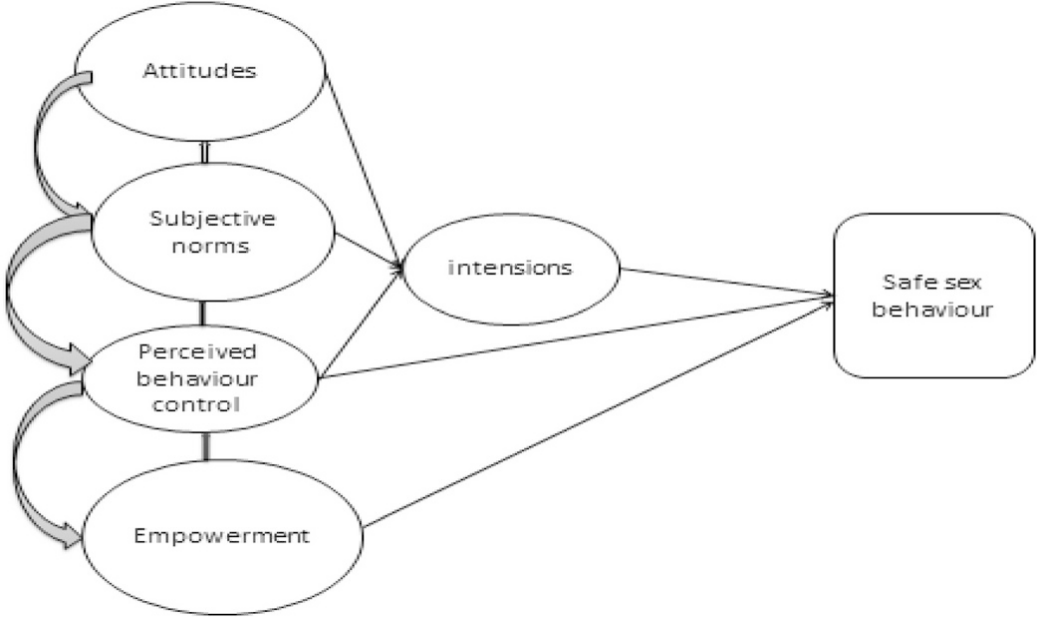
Theoretical model for this study (Adopted from Ajzen I [27] TPB constructs with addition of the empowerment component)

Guided by our conceptual framework (Fig. 1), we used Statistical package for Social sciences (SPSS) version 15 to analyse the collected data. Descriptive analysis of the background information was performed in order to get the initial picture of the studied population before progressing to detailed analysis. Age was operationalized as age in years and was split into two categories, distinguishing young adolescents (14–16 years) from older adolescents (17–19 years).Exploratory factor analysis using a principal components approach (PCA) was used to reduce the items to latent constructs and to obtain scales. Only items with a reliability coefficient (α) of at least 0.60 were considered acceptable [51]. Bivariate correlation (Pearson's correlation coefficient(r) was obtained in order to see how outcome variables relate to the scales obtained and also the background characteristics in the study. Furthermore, a diagnostic independent *t*-test was applied to check how the outcome variables relate to the obtained scales before conducting multivariate analysis. We employed logistic regression to determine the predictors of safe sex practices because our dependent variables were dichotomous. A backward selection procedure was applied to determine which independent (predictor) variables together were good predictors of each outcome in determining intention to practice safe sex and reported actual safe sex behaviour. The overall variance inflation factor (VIF) of the logistic regression model was 1.02, thus, indicating low degree of multicollinearity.

Since one of our major aims was to advance knowledge on condom use, in this study we considered only two outcome variables for regression analysis: (1) intention to engage in safe sex, that is, intention to use condoms during intercourse in the future and (2) reported actual safe sex behaviour (for the sexually active youth) that is use of condoms during the last sexual intercourse. The constructs from TPB and EHP were taken as predictors of safe sex behaviours.

### Ethical consideration

Ethical clearance was provided by the National Institute of Medical research (NIMR) Tanzania. After the permission from the District Executive Director, the District Education Officer and the Head Masters of the participating schools were asked for informed consent to permit the students to take part in the study. After being informed of the study, students were requested to sign for consent.

## Results

We present the results in accordance to respondents’ socio-demographics, sexual behaviours and determinants of intention to use condoms and reported use of condoms.

### Socio-demographics

Four hundred and three participants were recruited in this study of whom 50.4 % (203) were males and 49.6 % (200) were females .Two hundred and seventy seven (69 %) were in the age group 17–19 years. Although the mean age was 17.1 ± 1.44 years, males had slightly higher mean age (17.23 ± 1.40) than females (16.98 ± 1.47). The study participants were predominantly Muslims (87.1 %). Table 1 provides details of socio-demographic characteristics.

**Table 1:** Distribution of respondents by socio-demographic characteristics (*N* = 403)

*Socio-demographics*	*Frequency (%)*
*Sex*	
Female	203 (50.4 %)
Male	200 (46.9 %)
*Age (years)*	
14-16	126 (31 %)
17-19	277 (69 %)
Mean (Males) =17.23 ± 1.40	
Mean (Females) =16.98 ± 1.47	
Mean (Total) =17.1 ± 1.44	
*Religion*	
Moslems	351 (87.1 %)
Christian (Catholics)	29 (6.9 %)
Christian (others)	23 (6 %)

### Sexual behaviours

One hundred sixty four (40.6 %) of the participants were sexually active. More males (57.3 %) than females (42.7 %) were sexually active. Approximately eighty five percent of sexually active students reported to have started sex at age between 14 and 17 years and only 15.3 % started at an age less than 14 years. The majority of those who reported to be sexually active adolescents up to 84 % of them had a sexual encounter within 12 months prior to the survey.

Adolescents who reported that they had not used condoms at the last sexual intercourse were 78 (50.3 %) while those who used condoms were 49.7 %. There was no difference between males and females on the status of reported use of condoms at last sex (*p* = 0.284). Also, there was no significant difference between reported use of condoms among respondents with a single partner and those with multiple sex partners (Table 2).

**Table 2:** Distributions of sexual behaviours and social demographics

*Sexually active (regularly active)*	*Yes*	*No*	*Total*	*P-value*
*Gender*				
Male	57.30 %	42.20 %	50.40 %	
Female	42.70 %	57.80 %	49.60 %	0.23
Total (N)	164	239	403	
*Age (years)*				
14-16	15.20 %	43.20 %	31.80 %	0.266
17-19	84.80 %	56.80 %	68.20 %	
Total (N)	164	239	403	
*Number of lifetime sex partners* ^*^	*Yes*	*No*	*Total*	
One	49.30 %	55 %	52 %	0.019
Two or more	50.70 %	45 %	48 %	
Total (N)	81	83	164	
*Condom use at last sex* ^*^	*Yes*	*No*	*Total*	
*Gender*				
Male	62.80 %	53.80 %	58 %	
Female	37.70 %	46.20 %	42 %	0.284
Total (N)	81	83	164	
*Intention to use condoms*	*Yes*	*No*	*Total*	
Gender				
Female	50.90 %	41.20 %	51.1 %	0.148
Male	49.1	58.8	48.90 %	
Total (N)	306	97	403	
*Sexually activity (past 12 months)*				
Female	43.47 %	46.15 %	43.90 %	0.45
Male	56.53 %	53.85 %	56.10	
Total	138	26	164	

The majority of adolescents (77 %) in our sample indicated that they intent to use condoms in the future. There was no statistically significant difference between males and females on intention to use condoms in the future (*p* = 0.148). Two hundred and fifteen (70 %) adolescents aged 17–19 indicated that they intend to use condoms in the future, however there was no statistical significant difference with their counterparts (less than 17 years) (*p* = 0.266). Among sexually active adolescents, 78.7 % indicated future intensions to use condoms but there was no significant difference between current condom users and non-users on intentions to use condoms in the future (*p* = 0.071)

### Determinants of safe sex behaviours

Results of the binary analysis are presented in Table 3. Only perceived behaviour control showed a significant positive correlation with attitudes (r = 0.110, *p* < 0.05).

**Table 3:** Correlation of independent variables (*N* = 403)

Pearson correlation	Empowerment	Subjective norm	Perceived behaviour control	attitude
Empowerment	1			
Subjective norm	.004	1		
Perceived behaviour control	.075	.003	1	
attitudes	.080	.015	.110(**)	1

**p ≤ 0.05

Results in Table 4 show that empowerment was significantly associated with reported condom use (t = −2.348, *p* = 0.02). Perceived behaviour control was significantly associated with intention to use condoms (t = −5.813, *p* = 0.000) as seen in Table 5.

**Table 4:** Relationship between reported condom use with mean sum scores subjective norms, perceived behaviour control, attitudes and empowerment

Variable mean scores	Condom use at last sex (%) (*N* = 164)		t	*p*-value
	*Yes*	No		
Subjective norm	20.27	208	−1.105	0.296
Perceived behavior control	28.18	28.5	−0.454	0.651
Empowerment	22.9	24.9	−2.348	0.021
Attitudes	10.4	11.23	−1.451	0.149

**Table 5:** Relationship between intension to use condoms in the future with mean sum scores subjective norms, perceived behaviour control, attitudes and empowerment

Variable mean score	Intention to use condoms in the future (*N* = 403)		t	*p*-value
	Yes	No		
Subjective norm	20.54	19.84	1.854	0.064
Perceived behavior control	28.11	31.06	−5.813	0.000
Empowerment	24.2	24.2	−0.139	0.890
Attitudes	11.05	11.66	−1.382	0.160

Binary logistic regression results in Table 6 indicated that perceived behaviour control predicted intention to use condoms (AOR = 3.059, 95 % CI 1.324-7.065), which means, adolescents had a strong belief that they will use condoms in the future if they have sex. The results in in Table 7 indicate that empowerment (AOR = 3.694, CI 1.295-10.535) and positive attitude toward the use of condom (AOR = 3.484, CI = 1.132-10.72) were significantly associated with reported actual use of condoms.

**Table 6:** Crude and adjusted models for intentions to use condoms (*N* = 403*)*

	COR	*P*-value	95 % CI for OR	AOR	*P*-value	95 % CI (AOR
Empowerment	0.923	0.808	0.484 – 1.761	3.059	0.009	1.324 - 7.065
Perceived behaviour control	2.682	0	1.653 - 4.350			
Attitudes	1.275	0.309	0.798 - 2.035			
Knowledge	0.987	0.973	0.455 – 2.139			
Subjective norm	0.932	0.769	0.52 – 1.491

**Table 7:** Crude and adjusted for reported condom use among sexually active adolescents (*N* = 155)

	COR	*P*-value	95 % CI for COR	AOR	*P*-value	95 % CI for AOR
Empowerment	2.336	0.044	1.023 – 5.332	3.694	0.015	1.295-10.535
Perceived behaviour control	0.978	0.945	0.517 – 1.848	3.484	0.029	1.132-10.72
Attitudes	0.586	0.111	0.304 -1.131	0.196	0.01	
Knowledge	1.965	0.159	0.768 -5.027			
Subjective norm	1.054	0.87	0.560-1.983			

## Discussion

While a number of studies have been reported on adolescent sexual behaviour in Tanzania and around the Africa region, most were based on one theoretical model in understanding sexual behaviour. In the current study, an integrated approach to assessing sexual behaviour was sought by using both theoretical approaches that look at the individual and those which focus on the ecological environment of the adolescents residing in mostly rural areas of Tanzania. Specifically, this study investigated the practices of safe sexual behaviours among adolescents and the factors that predict intension to use condoms and reported actual use of condoms.

The results show that 40.6 % adolescents were sexually active. This finding is in concordance with existing evidence in other countries of around SSA [1, 52] A nationally representative study in Nigeria reported that only a fifth of adolescents (18 % males; 22 % females) were sexually active by the age of 19 years [53]. We also found that 49.8 % of sexually active youths had already had multiple sexual partners with females reporting more partners than males (*p* = 0.019). This proportion is higher than what was reported in Tanzania by another similar study [8]which reported a 15 % of sexually active adolescents had multiple partners. The setting being mostly rural and ways in which questions were designed in the present study could explain the difference observed. However, another study in rural Tanzania found that up to 42 % of sexually active adolescents had multiple sexual partners [6]. High prevalence of multiple sexual partners among sexually active adolescents have been reported elsewhere [6, 45] and in most cases, males reported more sexual partners than females [6, 8]

In the present study, it was also found that the prevalence of reported condom use stands at 50.30 % unlike existing evidence that shows that condom use can be as high as 73 % [1]. The low proportion of adolescents who reported to have used condom in the last sex to comparable studies might be due to factors such as negotiation skills to use a condom or unavailability of condoms when needed. Exavery and colleagues [6] found that 39 % of adolescents in rural Tanzania used condoms at last sex and attributed the lack of condom use to inequalities in condom availability as well as remoteness of some places in rural areas. Similar findings were reported in South Africa by Eaton and colleagues [4] that unavailability of condoms hindered youth to use condoms. Moreover, variation in cultural factors such as, gender roles and religion and differences of access to information, communication and education, might have contributed [54]

Correlation between predictor variables showed that only perceived behaviour control and attitudes are correlated but the correlation was not higher than 70 %. This finding indicates that other predictor variables were different from each other .It is also not surprising that perceived behaviour control correlates with attitudes as previous studies showed that perceived behaviour control may be similar to affective attitude [55]

We found that the reported actual use of condoms as an outcome variable was strongly determined by empowerment and attitudes. The finding explains the role of empowerment and positive attitudes of turning intentions into actions, but it might also mean that adolescents who are empowered and have positive attitudes towards condoms found it desirable to report that they used condoms in the last sex even when they did not (social desirability bias) as is always the case in self report data in sensitive topics [56].

In line with prior research that has indicated intention to use condoms to better predicted by perceived behaviour control than other TPB constructs and empowerment [30, 37], our findings then offer further support to the notion that intention to condom use is determined by perception of control of an individual towards condom use [57] .This might also mean that perceived behaviour control has a great importance on decision making to perform a certain behaviour whereas empowerment and positive attitudes appear to be important in turning intentions into viable actions. As such, future studies assessing condom use among adolescents in rural Tanzania can further investigate the combined effect of empowerment and attitudes on condom use using more rigorous designs.

Although this study was set to generate important evidence with regards to sexual behaviours of adolescents, some limitations need to be acknowledged. As a cross-sectional study, we cannot use the findings for causal relationships between variables. Response bias and social desirability bias was another limitation as data was collected through self-administered questionnaires.

Our findings may imply that health promotion programs that use a combined framework in designing safe sex promotion might have more impact than those that focus on Knowledge-Attitude–Practice or TPB socio-cognitive components alone. However, more rigorous studies might help to establish the effect and added value of the combined framework on safe sexual behaviours in these settings

## Conclusion

This study represents an initial attempt to examine the role of combined framework that takes into account socio-cognitive and ecological factors to study sexual behaviours among adolescents in rural Tanzania. Our findings suggest that a considerable proportion of adolescents in the study setting practice unsafe sexual behaviours. Perceived behaviour control predicted intentions to use condoms while empowerment and attitudes predicted reported condom use. Subjective norms had only indirect effects on intention and reported use of condoms. Application of combined framework that takes into account socio-cognitive factors as well as an ecological component can add value to safe sex promotion interventions. Moreover, community empowerment based on participatory frameworks that base on critical consciousness rising and influence on public health policy can add a value and support safe sex behaviour interventions that are aimed at penetrating community networks.

## Abbreviations

AOR: Adjusted Odds ratio
CI: Confidence Interval
COR: Crude Odds ratio
EHP: Empowerment Model of Health Promotion
HIV: Human Immunodeficiency Syndrome
NIMR: National Institute for Medical Research
PCA: Principal Component Analysis
SPSS: Statistical package for Social Sciences
SSA: Sub-Saharan Africa
TPB: Theory of Planned Behaviour
